# Anti-Proliferative Properties, Biocompatibility, and Chemical Composition of Different Extracts of *Plantago major* Medicinal Plant

**DOI:** 10.29252/ibj.25.2.106

**Published:** 2021-01-20

**Authors:** Samaneh Rahamooz-Haghighi, Khadijeh Bagheri, Hossein Danafar, Ali Sharafi

**Affiliations:** 1 Department of Plant Production and Genetics, Faculty of Agriculture, University of Zanjan, Zanjan, Iran;; 2 Zanjan Pharmaceutical Biotechnology Research Center, Zanjan University of Medical Sciences, Zanjan; Iran;; 3 Department of Pharmaceutical Biotechnology, School of Pharmacy, Zanjan University of Medical Sciences, Zanjan, Iran

**Keywords:** Gas chromatography-mass spectrometry, HCT-116 cells, Hemolysis, Lethal dose 50

## Abstract

**Background:**

To study the anticancer activity of *Plantago major*, we assessed the effect of ethanolic, methanolic and acetonic extracts of this plant on HCT-116, SW-480, and HEK-293 cell lines as control.

**Methods:**

The cytotoxic activity***, biocompatibility, and ***toxicity were evaluated by MTT assay, ***hemolysis, ***and *Artemia salina*-LD_50_ (on mice) tests, respectively. The analysis of the extracts was performed by GC-MS analysis.

**Results:**

The results showed that all the extracts had the most antiproliferative **properties** on the HCT-116 cell line. The *P. major* root extract was more effective than the aerial parts, and IC_50_ values for ethanolic, methanolic and acetonic root extracts were 405.59, 470.16, and 82.26 µg/mL, respectively on HCT-116 cell line at 72 h. Hemolysis degree of the ethanolic extract of aerial and root parts were approximately 1% at 400 μg/mL.. Using the ethanolic extracts, the *Artemia* survived every concentration, and no toxicity was observed. One week after the oral administration of different parts of *P. major *extracts, none of the mice died, even those were administered 2000 mg/kg. The results of GC/MS analysis showed that *P. major* extracts contain potential anticancer compounds, such as stearic acid (8.61%) in aerial parts of methanolic extract and 1,2- Benzenedicarboxylic acid, mono(2-ethylhexyl)ester (88.07% and 40.63%) in aerial and root parts of acetonic extract of *P. major*.

**Conclusions:**

Our findings suggest that the *P. major* is a source of potential compounds with antiproliferative properties.

## INTRODUCTION

Since the most anticancer drugs are associated with severe side effects, new drugs with higher efficacy and fewer adverse effects are needed^[^^1^^]^. Natural-based active compounds, such as curcumin, artemisinin, and taxol, have been used for cancer treatment^[^^2^^]^. Plant secondary metabolites, owing to their antiproliferative features, have also been utilized as drugs for the treatment of cancer^[^^3^^]^. 

*Plantago major *is a medicinal herb from the *Plantaginaceae*^[^^4^^]^. Based on traditional medicine, it has various medicinal uses without significant side effects. Most therapeutic effects of *P. major* described in traditional medicine have not fully been investigated; thus, complementary studies are required to reveal more medicinal characteristics of this plant. Having a broad range of medical application in many countries, *P. major* can be exploited as a source of cost-effective drug candidate^[^^5^^]^. *P. major *contains important compounds, such as polysaccharides, flavonoids^[^^6^^,^^7^^]^, phenolic compounds^[^^8^^]^, monoterpenoids, and triterpenoids^[^^9^^]^, lipids, and caffeic acid derivatives^[^^10^^]^. Moreover, caffeoyl phenylethanoid glycosides and iridoids glycosides are the most distinctive category of compounds that show important correspondence in their chemotaxonomy significance^[^^11^^-^^13^^]^. 

Terpenoids are relatively non-toxic substances; these compounds have great potential to be applied as chemotherapeutic agents in battling cancer^[^^14^^]^. As the main constituents of *Plantago* extract, triterpenoids have exhibited superb antiproliferative effects and apoptosis on cancer cells^[^^15^^-^^17^^]^. Fatty acids are one of the most important medicinal sources with multi-biological (antimicrobial and antifungal) activities^[^^18^^]^. 

Till now, various therapeutic effects of *P. major* have been revealed^[^^5^^]^. The antibacterial activity of *P. major* has been suggested in Holetz *et al*.’s study^[^^19^^]^. The aquatic extract of *P. major *has been indicated to have an effective antileukemia, anticarcinoma, and antiviral activities, along with modulate cell-mediated immunity^[^^20^^]^. Moreover, the aerial parts of this plant have been used for the treatment of anemia and hematopoietic disorders; however, its possessions on hematopoietic cells, mainly on totipotential stem cells, remain unidentified^[^^21^^]^. It has been reported that *P. major *L. has an uterotonic action in the guinea pig, a prophylactic influence on mammary cancer in mice, and a protective effect against systemic *Streptococcus pneumoniae *infection in mice^[^^22^^-^^24^^]^. *P. major* extract has a significant inhibitive effect on Ehrlich ascites tumor^[^^25^^]^. 

To the best of our knowledge there is no previous research, associated with the aerial and root parts of *P. major* on colon cancer cells (HCT-116 and SW-480 cell lines). Hence, we aimed to investigate the effect of the whole aerial parts and roots of this plant on two cell lines of colorectal cancer by MTT assay, hemolysis, toxicity activity on *Artemia salina* and LD_50_ determination, along with the analysis of the volatile compounds of this plant species. 

## MATERIALS AND METHODS

**Herbal materials**

The plants were collected from Zanjan city of Iran (geographical coordinates of the collection sites: 36°41'15.5"N 48°24'02.2"E) and authenticated at the Department of Botany, University of Zanjan, Iran. All the plant sections were cut into small pieces and dried in the shade at room temperature for one week. 

**Plant extraction**

Approximately 20 grams of the dried aerial (stem and leaf) and root parts of *P. major* was ground to a coarse powder and extracted by the reflux method using 200 mL of ethanol or methanol for 8 h and acetone for 3 hours. The extracts were then filtered and concentrated in an evaporator under pressure at 35-45 °C for 75 min. The extracts were kept at 4 °C^[^^26^^]^.

**Cell line culture**

HCT-116, SW-480, and HEK-293 cell lines were obtained from the Pasteur Institute of Iran, Tehran and were cultured in DMEM and RPMI-1640 media supplemented with penicillin-streptomycin (1%) and 10% FBS in 5% CO_2_ incubator at 37 °C.

***Viability assay***

The inhibitory effect of the ethanolic, methanolic and acetonic extracts of *P. major* on the cell lines was determined by MTT assay. The cells were seeded onto a 96-well plate at a density of 7 × 10^3^ cells/well. The cells were attached and grown for 24 h to reach 70-80% confluency. Subsequently, 10 mg of concentrated extracts was dissolved in 100 µL of DMSO and dissolved in 900 µL of the culture medium (DMEM or RPMI-1640) for the preparation of 25, 50, 100, 200, and 400 µg/mL of the extracts using dilution method. The extracts were filtered by 0.45-μm membrane filters. Medium and DMSO were considered as + and – control, respectively. Cells were treated with the prepared extracts and incubated for 1-3 days. Thereafter, 20 µL of MTT (5 mg/mL) was added and kept at an incubator for four ***hours****.* The media was then removed by aspiration. DMSO (200 µL) was added to each well to dissolve the obtained formazan. The absorbance was read by an ELISA plate reader (Tecan Infinite M200, Austria) at 570 and 690 nm, and the OD was documented^[^^27^^]^. The inhibitory rate of the cell growth was considered by: % Growth inhibition = (1 - OD extract-treated)/OD negative control × 100

**Hemolytic toxicity**

To examine the biocompatibility of the ***ethanolic extract of aerial and root parts,*** the hemolysis assay was performed^[^^28^^]^. Freshly prepared human RBCs collected in ethylenediaminetetraacetic acid-containing tube were washed with isotonic PBS (pH 7.4) by centrifugation at 1663 ×g for 5 min. Next, the tube containing the erythrocytes was resuspended in the same medium at a final hematocrit of 5%. Then aerial and root extracts with the concentration of 25, 50, 100, 200, and 400 μg/mL were added to 0.4 mL of diluted human RBC suspension. All of the samples were prepared in triplicate, and the suspension was shaken before incubation at 37 °C for 4 h. The sample was then centrifuged (Eppendorf Centrifuge 5417R) at 5400 ×g for 5 min to remove non-lysed human RBCs. Afterwards, 100 μL of the supernatant from the sample tube was moved to a 96-well plate. The supernatant was used, and hemoglobin release was assessed at 545 nm. Sodium dodecyl sulfate (0.1%) and PBS were used to establish 100% and 0% hemolysis as the positive and negative samples, respectively. The percentage of hemolysis was described by the following equation where + and - controls are the absorbance of the solution at 100 and 0% hemolysis. Hemolysis % = [(sample absorbance - negative control)/ (positive control - negative control)] ×100

**Toxicity assay on** ***A. salina***

The general toxicity of the ethanolic extracts on *A. salina* was assessed^[29]^. *A. salina* eggs were achieved from Urmia University, the West Azerbaijan Province, Iran. The cysts were seeded in a flask containing 35 g of NaCl in 1 L of distilled water. After incubation for 36-48 h at 28 °C, the larvae hatched within 48 h. The test was performed on the larvae of brine shrimp (*A. salina* Leach.). At first, a stock solution of 10 mg of ethanolic extract of aerial and root parts was dissolved in 100 µL of DMSO and then in 900 µL of the medium to prepare the stock (10 mg/ml). It was diluted to make the concentrations ranging from 0.78125 to 10 mg/ml. Ethanolic extracts (20 μL) was added to each well of the 96-well microtiter plates containing 180 μL of RPMI-1640 to form the extract concentration ranging from 1000 µg/mL to 7.8125 µg/mL. After that, 10 nauplii per well were added to the 96-well plates and incubated at 25 °C for 24 h. Afterwards, the numbers of surviving nauplii in each well were calculated under a binocular microscope after 24 h. All experimental settings for each concentration were in triplicates. Additionally, the negative control contained only 10 nauplii and artificial sea water. The percentages of nauplii deaths were calculated by considering the number of survivors in the test and control wells. The lethality was determined by Abbott’s formula: Lethality (%) = [(Test -ontrol)/Control] × 100.

**Oral acute toxicity**

An oral acute toxicity was performed to calculate the LD_50_. Ten Swiss Albino mice, obtained from the Pasteur Institute of Iran (25–35 g), were selected equally from both sexes. For the adaptation of the mice with laboratory conditions, every five mice were kept in a cage for seven days prior to testing and had free access to food and water according to OECD Guidelines with some modifications^[^^30^^]^. Different doses (control, 250, 500, 1000, and 2000 mg/kg) of the alcoholic extracts of *P. major* (various parts) were orally administered to each animal. If all animals were survived after 24 h, two additional mice were selected and treated at the highest dose (2000 mg/kg). If these two mice survived, then the LD_50_ was more than the limited dose, and the test was stopped. All tested animals were weighed before treatment and 24 h and one1 week after that. 

**GC-MS** **analysis**

GC-MS of the methanolic extracts (due to the better solubility of the compounds in methanol than ethanol) and acetonic extracts of *P. major *aerial and root parts was used for analysis. GC-MS analysis was carried out by Agilent technologies 5975c, USA. Next, 1 µL of the methanolic extract was subjected to the GC-MS system equipped with a capillary column (30 m × 250 µm × 0.25 µm, Agilent). Helium was used at the flow rate of 1.0 ml/min. The injector and the interface temperature were kept at 350 °C. The column temperature was attuned as follows: the initial temperature was 50 °C (2 min) then increased at a rate of 4 °C/min up to 230 °C (2 min). The identification of the components was determined by comparing mass spectral fragmentation patterns in MS data libraries (NIST08.L)^[^^31^^]^. 

**Statistical analysis**

The experiments were directed in triplicate, and group-wise comparison and statistical analysis of the results were performed by ANOVA and Duncan’s new multiple range test. SPSS v21 was used for statistical analyses. *p *< 0.05 was considered as statistically significant. The IC_50_ values were analyzed with ED50plus v1.0 software.

**Ethical statement**

The above-mentioned sampling/treatment protocols were approved by the Research Ethics Committee of University of Zanjan, Zanjan, Iran (ethical code: 21699). 

## RESULTS

**Cytotoxicity activity**

***Cell*** ***proliferation*** ***inhibition*** ***activity*** ***of different extracts of the P. major aerial parts***

**Fig. 1:**
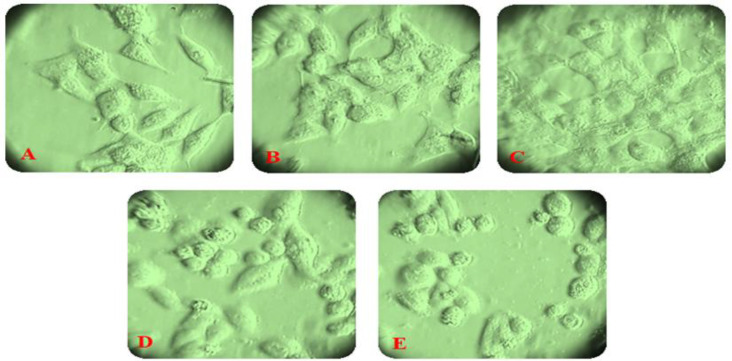
The growth inhibitory effect of acetonic extract of *P. major* aerial parts on the HCT-116 cancer cell line*.* )A( Cells were seeded at a density of 7 × 10^3^ cells per well in a 96-well plate. The morphology of cells after treatment with aerial parts acetonic extracts )B( one, )C) two, and (D and E) three days after treatment

**Fig. 2:**
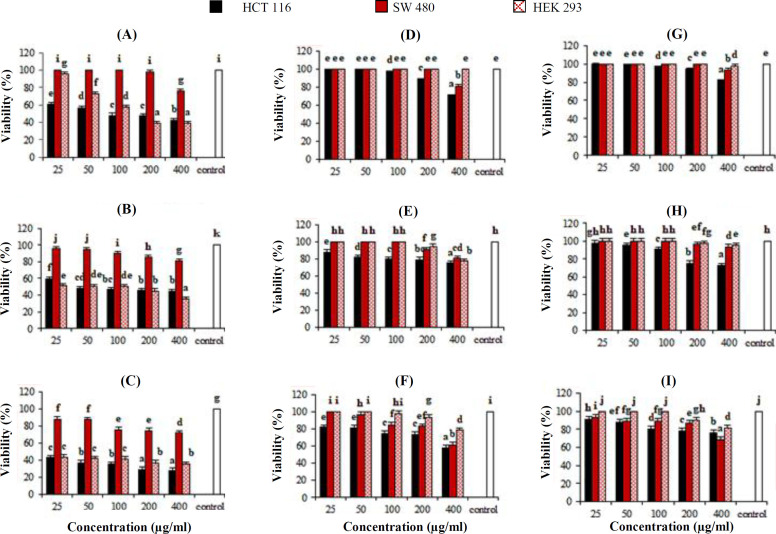
The result of MTT assay, one, two and three days after treatments. The percentage of the viability of the colorectal carcinoma cell lines (HCT-116 and SW-480) and embryonic kidney normal cell line (HEK-293) were treated with different extracts of aerial parts of *P. major* at 24, 48, and 72 h, respectively. The acetonic (A, B, and C), ethanolic (D, E, and F), and ethanolic (G, H, and I) extracts. Values represent the mean of three replications ± standard deviation

Based on the results, the alcoholic extracts of *P. major* represented more antiproliferative **properties** on the HCT-116 in comparison with SW-480 cell line (Fig. 1). Also, the acetonic extract of aerial parts had the most inhibition effect (59%, 43%, and 27% and 37%, 29%, and 23%, respectively) on the viability of HCT-116 cells and normal cell lines at the highest concentration (400 µg/mL) in 24, 48, and 72 h, (Fig. 2A, 2B, and 2C).

Despite the lower cytotoxicity of alcoholic and acetonic extracts on SW-480 cells, compared to HCT-116 cells, the alcoholic extracts showed 92-94% viability at the concentration of 400 µg/mL in 24 h, while acetonic extract had no effect at the same time and at 48 and 72 h. Besides, the acetonic extract had the same activity with alcoholic extracts on SW-480 cells. In both cell lines, the ethanolic extract had a better cytotoxicity effect than methanolic extract (Fig. D-2F and 2G-2I). This proliferation inhibition activity of the cells was not only time-dependent but also dose-dependent. The cytotoxic effect of the alcoholic extracts in comparison to acetonic extract on HEK-293 normal cells at 72 h showed that the alcoholic extracts had low inhibitory effects (between 2% to13%) only at the concentrations of 200 and 400 µg/mL, whereas the acetonic extract revealed a significant cytotoxicity effect on the HEK-293 cells in all tested concentrations (25-400 µg/mL). Indeed, these results indicated that the alcoholic extracts of aerial parts of *P. major* had significant cytotoxic activity on cancer cell lines, whereas at high concentration, it showed a partial cell proliferation inhibition activity on normal cells. However, the acetonic extract possessed high cytotoxicity activity on both cancer and normal cell lines; therefore, based on the results, the use of acetonic extract is not recommended for the **treatment of** colorectal cancer.

IC_50_ values of ethanolic, methanolic and acetonic extracts were calculated as follows: 475.20, 655.09, and 221.64 μg/mL for HCT-116 cells, 646.06, 756.38, and 715.28 μg/mL for SW-480 cells, and 904.98, 1016.55, and 107.85 μg/mL for HEK-293 cell line at 72 h, respectively (Table 1). The lowest IC_50_ was attributed to the effect of the acetonic extract on HCT-116 cells (221.64 μg/mL) and HEK-293 cells (107.85 μg/mL).

***Cell*** ***proliferation*** ***inhibition*** ***activity*** ***of root part*** ***extracts of the P. major ***

**Fig. 3:**
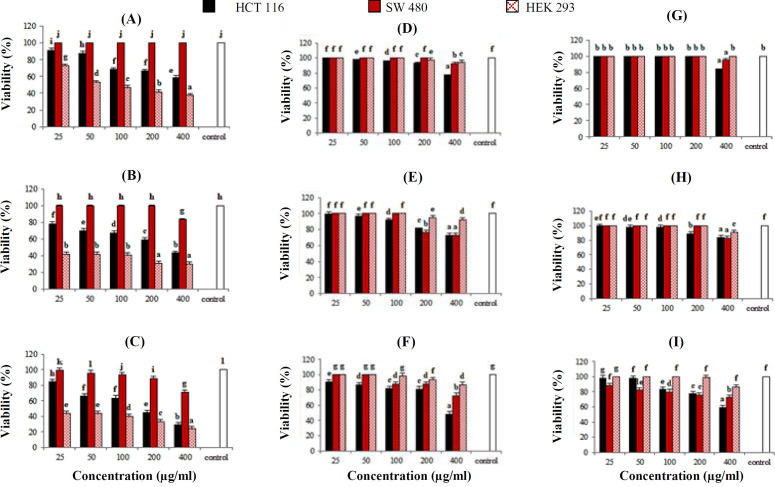
The result of MTT assay, one, two and three days after treatments. The percent of the viability of the colorectal carcinoma cell lines (HCT-116 and SW-480) and embryonic kidney normal cell line (HEK-293) were treated with different extracts of the root part of *P. major* at 24, 48, and 72 h, respectively. The acetonic (A, B, and C), ethanolic (D, E, and F), and methanolic (G, H, I) extracts. Values represent the mean of three replications ± standard deviation

The *P. major* (ethanolic, methanolic and acetonic) root extracts had more cytotoxic activity on the HCT-116 than SW-480 cells, similar to the aerial parts extracts (Fig. 3A-3C); however, the *P. major* root extracts had more antiproliferative **activity than **aerial parts extract** on SW-480 cells** (Fig. 3D-3F).** Although the methanolic extract was found to have less **antiproliferative **properties than the ethanolic extract** **of *P. major* root part, the root methanolic extract displayed a more cytotoxic effect on HCT-116 than aerial parts extracts **(Fig. 3G-3I). **The inhibitory effect of acetonic extracts *P. major* root on the viability of HEK-293 normal cells was considerable (65%). This effect on root extracts was less than the extracts obtained from aerial parts (76%) at 72 h, **despite its greater cytotoxic effect on cancer cell lines. IC_50_ values of ethanolic, methanolic and acetonic extracts were 405, 470, and 82 µg/mL for HCT-116 cells, 513, 687, and 698 µg/mL for SW-480 cells, and 948, 1563, and 125 µg/mL for HEK-293 cells at 72 h, respectively (Table 1). The lowest IC_50_ was related to the effect of the acetonic extract on HCT-116 cells and HEK-293 cells. Since the IC_50_ values of the ethanolic extract on HCT-116 and SW-480 cell lines (405 and 513µg/mL) were less than a normal cell (948 µg/mL), this extract possessed valuable characteristics. The IC_50_ values of the alcoholic and acetonic extracts of *P. major* root on cancer cell lines were lower than the aerial parts extracts. 

**The biocompatibility** assay **of the ethanolic extracts** (hemolysis** toxicity)**

**Fig. 4:**
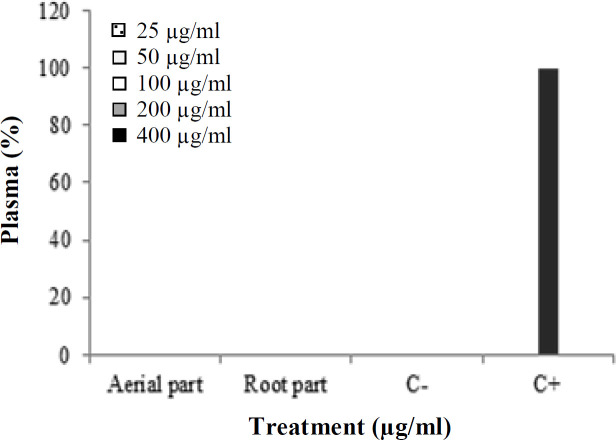
Hemolytic assay to verify the biocompatibility of ethanolic extracts of *P. major* aerial and root parts

To verify the biocompatibility of the ethanolic extracts of *P. major* aerial and root parts, we examined the hemolytic activity. Hemolysis degree for both the aerial and root parts of *P. major* was determined to be lower than 1% at the highest concentration (400 μg/mL) for 4 h (Fig. 4). 

**Toxicity assay of ethanolic extracts on** ***A*** ***.*** ***salina***

**Fig. 5:**
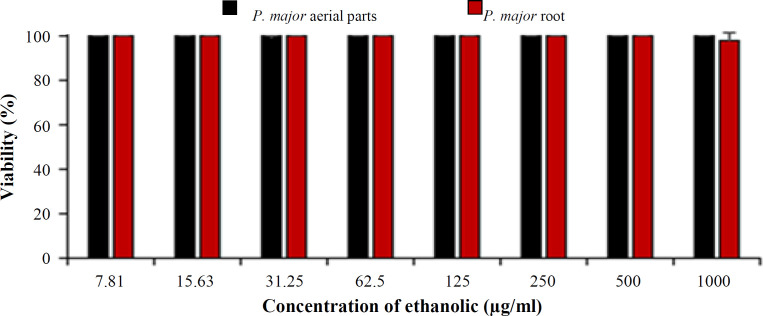
Toxicity of ethanolic extracts of *P. major* on *A. salina*

The general toxicity of aerial and root parts of *P. major* ethanolic extracts was assessed against *A. salina*. The percentage of lethality was used as a bioassay indicator for the toxicity of ethanolic extracts. At all concentrations of ethanolic extracts ranging from 7.8125 to 1000 μg/mL, all of the nauplii were alive, and no toxicity was observed (Fig. 5). 

**Oral acute toxicity assay of the alcoholic extract (LD**_50_ **)**

Mice were treated with a range of oral doses. Acute toxicity is known as an *in vivo* biocompatibility test. One week after the oral administration of *P. major* aerial and root parts extracts, none of the animals were died. According to OCED and Hodge and Sterner Scale, we can approve that both the aerial and root extracts of *P. major* were practically safe and non-toxic. Furthermore, the change in the body weight is an important factor and an indicator of the potential toxicity (Table 2). The weight of mice after 24 and 168 hours (one week) faintly increased, indicating that all the mice had a natural activity with normal behavior.

**Determination of the composition of alcoholic and acetonic extracts by GC-MS**

The composition of different extracts of *P. major *was evaluated by GC-MS, and its components were identified by the NIST08.L library. The extracts of *P.* *major* showed the presence of fatty acids, phenols, terpenoids, amines, amides, siloxanes, esters, alkanes, aldehydes, benzene derivatives etc. The presence of the volatile components were carried out by GC-MS, which detected acetol (5.03%), elaidic acid (5.48%), octacosane (6.12%), octadecanoic acid (8.61%), and palmitic acid (15.18%) compounds in the analysis of the methanolic extract of the *P. major* aerial part. However, in the acetonic extracts, it found Bis(2-ethylhexyl) phthalate (3.67%), 1,2-Benzenedi-carboxylic acid, and mono(2-ethylhexyl) ester (88.07%). GC-MS also detected octasiloxane, 1,1,3,3,5,5,7,7,9,9,11,11,13,13, 15,15-hexadecamethyl- (4.97%), cyclohexasiloxane, dodecamethyl- (6.35%), verbenone (6.96%), isoborneol (8.68%), tetradecamethylcycloheptasiloxane (9.74%), and n-hexadecanoic acid (13.8%) in the methanolic extract of *P. major* root part and heneicosane (5.23%), cis-9-hexadecenoic acid (7.99%), n-hexadecanoic acid (9.88%), 13-Docosen-1-ol, (Z)- (15.91%) 1,2-benzenedicarboxylic acid, and mono(2-ethylhexyl) ester (40.63%) in the acetonic extracts (as the dominant constituents). A list of compounds identified by GC-MS analysis is represented in Supplementary Table 3. In the present study, the common compounds in the methanolic extracts of both parts of the plant were benzaldehyde, 2-nitro-,diaminomethylidenhydrazone (0.25% and 1.40%), pentadecanoic acid, 13-methyl-,methyl ester (0.36% and 1.76%), hexadecamethylcyclo-octasiloxane (0.32% and 4.51%), and hexadecanoic acid (15.17% and 13.79%); however, in the acetonic extracts, the most frequent compounds were 1,2-benzenedi-carboxylic acid, mono(2-ethylhexyl) ester (88.07% and 40.63%), 2-pentanone, 4-hydroxy-4-methyl- (0.12%, and 1.29%), and Oxirane,heptadecyl- (1.01% and 1.98%), respectively. Both cyclohexasiloxane dodecamethyl-/CAS number: 540-97-6 (1.71%, 6.35%, 0.89%, and 0.47%) and cycloheptasiloxane, tetra-decamethyl-/CAS number: 107-50-6 (1.16%, 9.74%, 1.02%, and 1.68%) compounds were cyclic methyl siloxanes and found in both the methanolic and acetonic extracts of aerial and root parts of the plant with different percentage, respectively.

**Table 1:** IC_50_ estimations of various extracts on HCT-116, SW-80, and HEK-293 cell lines at different exposure times

**Cell/part**	**24 h**			**48 h**			**72 h**		
	**Methanolic**	**Ethanolic**	**Acetonic**	**Methanolic**	**Ethanolic**	**Acetonic**	**Methanolic**	**Ethanolic**	**Acetonic**
HCT-116									
Aerial	1391.12 ± 3.1	930.87 ± 2.3	436.76 ± 2.5	1170.76 ± 1.8	698.60 ± 3.1	307.34 ± 4.5	655.09 ± 1.9	475.20 ± 3.0	221.64 ± 2.2
Root	1142.78 ± 1.7	897.14 ± 4.1	229.91 ± 2.4	655.09 ± 4.2	897.46 ± 3.6	220.12 ± 2.8	470.16 ± 2.5	405.59 ± 3.4	82.26 ± 2.7

SW-480									
Aerial	4065.77 ± 2.3	2877.36 ± 2.4	3876.00 ± 4.8	2822.86 ± 2.2	990.24 ± 3.0	1355.71 ±3.4	756.38 ± 2.6	646.06 ± 3.5	715.283 ± 3.4
Root	3152.87 ± 2.4	1173.53 ± 3.1	1054.53 ± 1.4	1275.02 ± 5.1	713.23 ± 1.3	901.53 ± 1.2	687.12 ± 3.3	513.08 ± 3.4	698.19 ± 1.8
									
HEK-293									
Aerial	3877.36 ± 9.2	3353.17 ± 2.3	204.70 ± 3.7	2366.70 ± 4.1	2381.27 ± 1.4	107.82 ± 2.5	1016.55 ± 3.8	904.98 ± 2.6	107.85 ± 2.7
Root	8824.49 ± 3.6	4026.77 ± 5.8	246.42 ± 1.9	4202.72 ± 4.2	1443.96 ± 2.4	184.76 ± 4.3	1563.04 ± 1.3	948.15 ± 3.9	125.89 ± 1.5

ED_50_ plus V1.0 gives an estimated value of IC_50_; Mean of three replications ± SD at 24, 48, and 72 h.

## DISCUSSION

*P. major *is a medicinal plant with various therapeutic benefits. *P. major* extract can inhibit the cell proliferation of melanoma, renal and mammae^[^^32^^]^, skin^[^^10^^]^, leukemia^[^^33^^]^, breast adenocarcinoma, and melanoma (UACC-62) cell lines^[^^16^^]^. The *in vitro* cytotoxic activity of the methanolic extract of *P. major *has been evaluated on HCT-15, SQC-UISO, OVCAR, and KB^[^^21^^]^, which demonstrated that *P. major *has hematopoietic activity. A comprehensive literature review on *P. major* extract, as an antineoplastic agent, revealed that this plant is an efficient chemoprophylactic and antimetastatic agent against several malignancies, such as breast cancer, hepatoma, and Ehrlich ascites tumor^[^^23^^,^^34^^-^^36^^]^. Based on the research conducted in 2013, *P*.* major* extract displayed a proapoptotic impact on the hypergastrinemia rat (*Rattus norvegicus*) model. This effect has been attributed to the increased level of caspase 3^[^^37^^]^. Cytotoxicity potential of methanolic extracts from leaves of *P. major *were evaluated against three human cancer cell lines. The results indicated that *P. major *extracts have cell proliferation inhibition activity on breast adenocarcinoma and melanoma cell lines recommended by the National Cancer Institute (USA)^[^^16^^]^.

Considering the aforementioned properties of *P. major*, in the first phase of the current study, the cytotoxic effects of the different extracts of *P. major *was examined by MTT test on HCT-116, SW-480, and HEK-293. The ethanolic, methanolic and acetonic extracts of *P. major* exhibited a significant cytotoxic activity on colorectal carcinoma cell lines. Based on the obtained results, the ethanolic extract was more cytotoxic compared to the methanolic extract and did not show any significant cytotoxicity effect on the normal cell line, except for in the concentrations of 200 and 400 µg/mL. In this regard, the alcoholic extracts derived from the root parts of *P. major *might be deliberated as a valuable source of metabolites with potential uses as antitumor drug precursors. The US National Cancer Institute argues that the IC_50_ value should be below 30 µg/mL so that a crude extract can serve as an appropriate agent for further refinement^[^^2^^]^. The root extracts of *P. major* showed more cytotoxicity than the aerial parts (leaf and stem) extracts and had a lower and more valuable IC_50_ index at 72 h. Indeed, these results indicated that the alcoholic extracts of aerial parts of *P. major* had significant cytotoxic activity on cancer cell lines, whereas at high concentration, it showed a partial cell proliferation inhibition activity on normal cells. However, the acetonic extract possessed high cytotoxicity activity on both cancer and normal cell lines; therefore, using acetonic extract is not recommended for the **treatment **of colorectal cancer. Since the IC_50_ value of the ethanolic extract on HCT-116 and SW-480 cell lines (475.20 and 646.06 µg/mL) was less than a normal cell (904.98 µg/mL); hence, it can be considered as valuable and useful extracts for medicinal treatment. 

In the second phase of the current study, aerial and root parts of *P. major* extracts were evaluated by hemolysis assay, general toxicity assay on *A. salina *and oral acute toxicity study (LD_50_) on adult Swiss Albino mice. The study of Atta *et al.*^[^^38^^]^ has reported that the oral administration of *P. major* seed methanol extracts in doses up to 2.5 g/kg body weight did not cause any major signs of acute toxicity, and no deaths were reported up to 72 h after the oral administration^[^^38^^]^. In this study, at all the concentrations of extracts, we observed no toxicity. Hence, it confirms the safety of these extracts and shows that these extracts are practically nontoxic. Mirzaei *et al.*^[^^39^^]^ selected the *P. major *to investigate its toxicity against both *A. salina* and *A. uramiana. *The result showed a positive correlation between the data obtained from the two aforesaid species, and the LC_50 _of the *P. major *was 303.7 μg/ml for the methanolic extract, whereas the thymol standard possessed the LC_50_ value of 7.2 μg/ml. 

**Table 2:** Weight change and Mortality rates of mice after 24 hours and one week, submitted to various doses of  *P. major*

**Groups**	**Dose** **(mg/kg)**	**No. of animals**	** Mean weight of** ** animals (24 h)**	**Duration of study (one week)**	**Dead rate** **(%)**
Control	-	5	30 ± 1.19	34 ± 1.11	**0**
M. A. M	175	5	35 ± 1.10	36 ± 1.27	0
	1750	5	38 ± 1.27	37 ± 1.58	0
	5000	5	29 ± 1.58	31 ± 1.30	0
M. A. E	17.5	5	30 ± 1.21	31 ± 1.27	0
	175	5	35 ± 1.64	34 ± 1.15	0
	1750	5	39 ± 2.23	38 ± 1.45	0
	5000	5	25 ± 0.70	28 ± 1.33	0
M. R.M	17.5	5	33 ± 2.05	33 ± 1.58	0
	175	5	35 ± 1.42	37 ± 1.58	0
	1750	5	39 ± 2.23	39 ± 1.76	0
	5000	5	25 ± 1.45	26 ± 0.79	0
	17.5	5	35 ± 1.33	34 ± 2.57	0
M. R. E	175	5	36 ± 2.91	37 ± 0.79	0
	1750	5	36 ± 1.27	34 ± 4.69	0
	5000	5	24 ± 1.27	24 ± 0.70	0

Value is mean ± standard deviation. M.A.M and M.A.E., *P. major* aerial parts methanolic and ethanolic extracts, respectively; M.R.M. and M.R. E., *P. major* root methanolic and ethanolic extracts, respectively.

Indeed, it can be concluded that this extract did not exhibit high toxicity in comparison with the thymol. Thus, this medicinal plant can be considered as a safe and non-toxic agent^[^^39^^]^. This result showed that the high concentration of both aerial and root parts of *P. major* did not affect the membrane integrity of RBCs and both *P. major *aerial and root extracts are biocompatible.It is interesting to note that the hemolytic activity of *P. major *has not been reported so far, and the obtained data indicated that this medicinal plant is practically nonhemolytic since its hemolysis value is below 1%. It also been reported that when the hemolysis percentage does not exceed 10%, it can serve as a non-toxic agent^[^^40^^]^. Moreover, we performed GC-MS to investigate the components of the extracts of aerial and root parts of *P. major*. The GC-MS analysis of extracts from *P. major* revealed the existence of fatty acids, terpenoids, siloxanes, and other medicinal compounds. Therefore, the extracts can be used as anticancer drugs against tumor growth and propagation because *P. major* has effective anticancer compounds. Some of the compounds identified in our study of analysis of the* P. major* extracts include Silanediol, dimethyl-^[^^41^^]^, camphor^[^^42^^]^, Borneol^[^^43^^]^, Isoborneol/Isocamphol^[^^44^^]^, 1,2-Benzenedicarboxylic acid, and mono(2-ethylhexyl) ester with antiviral features^[^^45^^]^. Two antimalarial compounds, i.e. 2,4-Di-tert-butylphenol^[^^46^^]^ and n-hexadecanoic acid^[^^47^^]^, were also found in the root extracts. Nortriptyline is a commercially available compound that has anti-depressant properties^[^^48^^]^; however, most of the compounds reported in the extracts have antibacterial, antifungal, anticancer, antioxidant and anti-inflammatory properties and are fatty acid. Fatty acids, such as oleic acid, linoleic acid, and palmitic acid, are important sources with antimicrobial and antifungal activities^[^^18^^]^. The antimicrobial properties of *P. major *are attributed to the presence of camphor, bornyl acetate, and borneol compounds; the biological activities of these compounds have previously been investigated^[^^49^^]^.

Our findings support the notion that the extracts of *P. major* might contain a variety of secondary metabolites that represent the multi-biological activities, which can be applied for the development of antitumor drug precursors. The purification of these bioactive compounds is thought to be useful for the formulation of therapeutic agents against cancer. Some of the compounds identified in this study, including, 2,4-Di-tert-butylphenol, camphor, gentisic acid, isoborneol, leinoleic acid, methyl ester/linoleic acid ester, myristic acid, methyl sterate, oleic acid, palmitic acid, p-cymene, stearophanic acid, trans-anethole etc., are therapeutically important.

## Supplementary Materials

Supplement
