# Supplementary material for: Anti-Proliferative Properties, Biocompatibility, and Chemical Composition of Different Extracts of Plantago major Medicinal Plant
Source: Iran Biomed J. 2021 Jan 20;25(2):106–16. doi: 10.29252/ibj.25.2.106 (PMC7921524; doi:10.29252/ibj.25.2.106)
Supplement: Supplement [file ibj-25-106-s001.pdf]

Table 3. Identified compounds in root part of *P.major* acetonic extract by GC-MS analysis

RT	Area Pct	Library/ID	Formula	M.w (g/mol)	Biological Activity	Nature of compound	Structure of compounds
5.1697	1.8469	Succinic acid, heptyl 2-methoxyethyl ester	$C_{14}H_{26}O_5$	274.35	No activity found	Oxygenated compounds	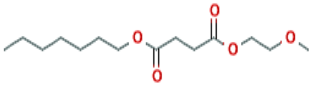
5.3185	1.2951	2-Pentanone, 4-hydroxy-4-methyl-/ Diacetone alcohol	$C_6H_{12}O_2$	116.16	Diacetone alcohol is found in fruits	Beta-hydroxy ketone,	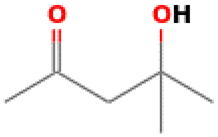
20.8307	0.4717	Cyclohexasiloxane, dodecamethyl-	$C_{12}H_{36}O_6Si_6$	444.92	Emollient, Personal care products, Lubricant and de-foaming agent, Antioxidant	Cyclic methyl siloxane	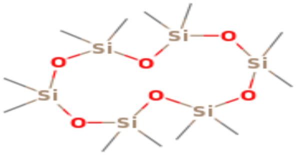
26.2151	1.6885	Cycloheptasiloxane, tetradecamethyl-	$C_{14}H_{42}O_7Si_7$	519.07	Antifungal, Skin-Conditioning Agent, Fragrance, Antimicrobial, Antifouling, Imunomodulatory, Antitumor	Cyclic methyl siloxane	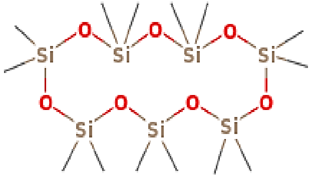
37.3959	0.7034	Methyl hexadec-9-enoate /Methyl palmitelaidate	$C_{17}H_{32}O_2$	268.4348	No activity found	Esters	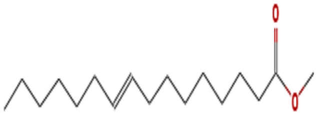
37.682	1.1641	Hexadecanoic acid, methyl ester/ Palmitic acid methyl ester	$C_{17}H_{34}O_2$	270.5	Antioxidant, Hypocholesterolemic, Nematicide, Pesticide, Antiandrogenic, Flavor, Hemolytic, Antibacterial, Antifungal	Fatty acid methyl ester	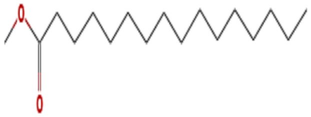

RT	Area Pct	Library/ID	Formula	M.w (g/mol)	Biological Activity	Nature of compound	Structure of compounds
38.6089	7.9933	cis-9-Hexadecenoic acid/cis-palmitic acid	$C_{16}H_{30}O_2$	254.4082	No activity found	Monounsaturated Fatty acid	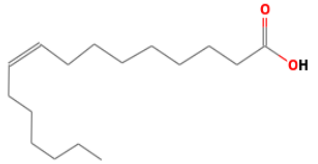
38.8664	9.8877	n-Hexadecanoic acid /Palmitic acid	$C_{16}H_{32}O_2$	256.42	Antioxidant, Pesticide, Antifibrinolytic, Hemolytic, Nematicide, Antialopecic, Antimicrobial, Antifungal, Hypocholesterolemic, Antiandrogenic	Fatty acid	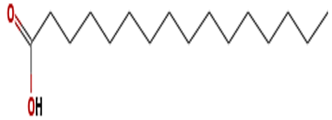
41.0923	0.7426	10-Heneicosene (c,t)	$C_{21}H_{42}$	294.6	No activity found	Not determined	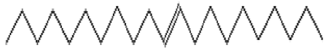
41.6301	0.5364	9,12-Octadecadienoic acid, methyl ester	$C_{19}H_{34}O_2$	294.4721	Hepatoprotective, Anti-histaminic, Antieczemic, Hypocholesterolemic, Antioxidant, Antimicrobial	Fatty acid methyl ester	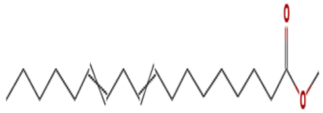
41.7159	5.2327	Heneicosane/ Eicosane, methyl-	$C_{21}H_{44}$	296.6	Antimicrobial agents	Aliphatic alkanes	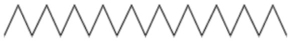
42.3511	0.5563	Octadecanoic acid, methyl ester /Methyl sterate	$C_{19}H_{38}O_2$	298.5	Antimicrobial, anti-inflammatory, Anticancer	Fatty acid methyl ester	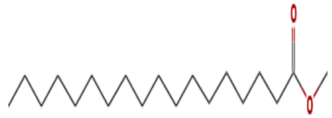
42.8317	3.6286	9,12-Octadecadienoic acid (Z,Z)-/ Linoleic acid/ Leinoleic acid	$C_{18}H_{32}O_2$	280.4455	Antimicribial, Antifungal, Hepatoprotective, Anti-histaminic, Anti-inflammatory, Cancer preventive, insectifuge, Nematicide, Antioxidant	Triterpenoids	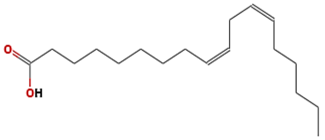
43.3925	0.6646	Octadecanoic acid/ stearic acid/Stearophanic acid	$C_{18}H_{36}O_2$	284.5	Antimicrobial	Saturated fatty acids	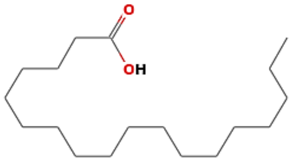

RT	Area Pct	Library/ID	Formula	M.w (g/mol)	Biological Activity	Nature of compound	Structure of compounds
46.0246	0.8746	Eicosane/ Nonadecane, methyl-	$C_{20}H_{42}$	282.5	Antifungal, Antitumor, Anticancer, Antibacterial	Aliphatic alkanes	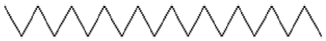
46.6426	0.9524	Methyl 18-methylnonadecanoate	$C_{21}H_{42}O_2$	326.6	No activity found	Esters	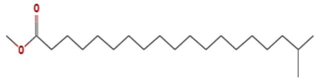
48.857	3.2329	Oxirane, hexadecyl-/1,2-Epoxyoctadecane	$C_{18}H_{36}O$	268.4778	No activity found	Not determined	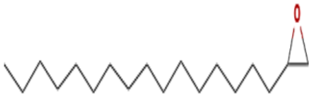
51.6664	1.9847	Oxirane, heptadecyl-/Heptadecyloxirane	$C_{19}H_{38}O$	282.5	No activity found	Ethylene oxides	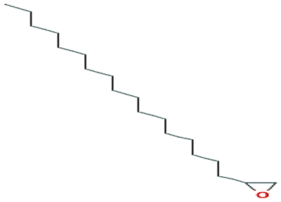
52.3645	40.6303	1,2-Benzenedicarboxylic acid, mono(2-ethylhexyl) ester	$C_{16}H_{22}O_4$	278.3435	Antimicrobial, Cytotoxicity, Antioxidant, Anti-inflammatory, Antiviral	Aromatic dicarboxylic ester	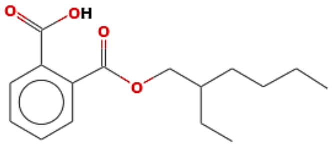
55.3571	15.9131	13-Docosen-1-ol, (Z)-	$C_{22}H_{44}O$	324.6	No activity found	Monounsaturated alcohol	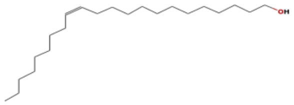
